# Supplementary material for: Can Quality Improvement Methodologies Derived from Manufacturing Industry Improve Care in Cardiac Surgery? A Systematic Review
Source: J Clin Med. 2022 Sep 12;11(18):5350. doi: 10.3390/jcm11185350 (PMC9502537; doi:10.3390/jcm11185350)
Supplement: Supplementary file 1 [file jcm-11-05350-s001.zip › jcm-1897285-supplementary.pdf]

Supplementary File

Legend

Supplementary material

Supplementary Table S1: Search Strategy

Supplementary Table S2: PRISMA checklist

Supplementary Table S1: Search strategy

PubMed (251)

Search	Query	Results
#4	#3 NOT ("Adolescent"[Mesh] OR "Child"[Mesh] OR "Infant"[Mesh] OR "adolescen*"[tiab] OR "child*" [tiab] OR "schoolchild*"[tiab] OR "infant*"[tiab] OR "girl*"[tiab] OR "boy"[tiab] OR "boys"[tiab] OR "teen*"[tiab] OR "youth*"[tiab] OR "pediatr*"[tiab] OR "paediatr*"[tiab] OR "pube*"[tiab])	251
#3	#1 AND #2	297
#2	"Total Quality Management"[Mesh] OR "six sigma*"[tiab] OR "statistical process control"[tiab] OR "root cause analys*"[tiab] OR "total quality management*"[tiab] OR "value stream mapping*"[tiab] OR "process improvement*"[tiab] OR "process assessment*"[tiab] OR ‘‘TQM’’[tiab] OR ‘‘toyota production system*’’[tiab] OR ‘‘motorola production system*’’[tiab] OR ‘‘henry ford production system*’’[tiab] OR ‘‘6 sigma’’[tiab] OR ‘‘lean proces*’’[tiab] OR ‘‘lean thinking’’[tiab] OR ‘‘lean method*’’[tiab] OR ‘‘lean sigma’’[tiab] OR ‘‘kaizen’’[tiab] OR ‘‘muda’’[tiab] OR ‘‘PDSA’’[tiab] OR "Plan-Do-Study-Act"[tiab] OR "Plan-Do-check-Act"[tiab] OR ‘‘PDCA’’[tiab] OR ‘‘continuous quality improvement*’’[tiab] OR ‘‘CQI’’[tiab] OR ‘‘statistical quality control*’’[tiab] OR ‘‘statistical process control*’’[tiab] OR ‘‘SQC’’[tiab] OR ‘‘SPC’’[tiab] OR ‘‘control chart*’’[tiab] OR ‘‘pareto chart*’’[tiab] OR "DMAIC"[tiab] OR "Process mapping"[tiab] OR "clinical audit*"[tiab]	29,635
#1	"Cardiac Surgical Procedures"[Mesh] OR "Heart/surgery"[Mesh] OR "cardiac surg*"[tiab] OR "cardiac operat*"[tiab] OR "cardiac preoperat*"[tiab] OR "cardiac intraoperat*"[tiab] OR "cardiac postoperat*"[tiab] OR "cardiac perioperat*"[tiab] OR "heart surg*"[tiab] OR "heart operat*"[tiab] OR "heart preoperat*"[tiab] OR "heart intraoperat*"[tiab] OR "heart postoperat*"[tiab] OR "heart perioperat*"[tiab]	281,252

Embase (811)

Search	Query	Results
#6	#4 NOT #5	811
#5	#4 AND ('conference abstract'/it OR 'conference paper'/it)	388
#4	#3 NOT (('juvenile'/exp OR 'embryo'/exp OR 'fetus'/exp OR (‘adolescen*’ OR ‘child*’ OR ‘schoolchild*’ OR ‘infant*’ OR ‘girl*’ OR ‘boy’ OR ‘boys’ OR ‘teen*’ OR ‘youth*’ OR ‘pediatr*’ OR ‘paediatr*’ OR ‘pube*’):ti,ab,kw))	1,199
#3	#1 AND #2	1,522
#2	'total quality management'/exp OR (‘six sigma*’ OR ‘statistical process control’ OR ‘root cause analys*’ OR ‘total quality management*’ OR ‘value stream mapping*’ OR ‘process improvement*’ OR ‘process assessment*’ OR ‘TQM’ OR ‘toyota production system*’ OR ‘motorola production system*’ OR ‘henry ford production system*’ OR ‘6 sigma’ OR ‘lean proces*’ OR ‘lean thinking’ OR ‘lean method*’ OR ‘lean sigma’ OR ‘kaizen’ OR ‘muda’ OR ‘PDSA’ OR ‘Plan-Do-Study-Act’ OR ‘Plan-Do-check-Act’ OR ‘PDCA’ OR ‘continuous quality improvement*’ OR ‘CQI’ OR ‘statistical quality control*’ OR ‘statistical process control*’ OR ‘SQC’ OR ‘SPC’ OR ‘control chart*’ OR ‘pareto chart*’ OR ‘DMAIC’ OR ‘Process mapping’ OR ‘clinical audit*’):ti,ab,kw	96,637
#1	'heart surgery'/exp OR (‘cardiac surg*’ OR ‘cardiac operat*’ OR ‘cardiac preoperat*’ OR ‘cardiac intraoperat*’ OR ‘cardiac postoperat*’ OR ‘cardiac perioperat*’ OR ‘heart surg*’ OR ‘heart operat*’ OR ‘heart preoperat*’ OR ‘heart intraoperat*’ OR ‘heart postoperat*’ OR ‘heart perioperat*’):ti,ab,kw	421,837

Clarivate Analytics/Web of Science Core Collection (399)

Search	Query	Results
#4	#3 NOT TS=("adolescen*" OR "child*" OR "schoolchild*" OR "infant*" OR "girl*" OR "boy" OR "boys" OR "teen*" OR "youth*" OR "pediatr*" OR "paediatr*" OR "pube*")	399
#3	#1 AND #2	478
#2	TS=("six sigma*" OR "statistical process control" OR "root cause analys*" OR "total quality management*" OR "value stream mapping*" OR "process improvement*" OR "process assessment*" OR ''TQM'' OR ''toyota production system*'' OR ''motorola production system*'' OR ''henry ford production system*'' OR ''6 sigma'' OR ''lean proces*'' OR ''lean thinking'' OR ''lean method*'' OR ''lean sigma'' OR ''kaizen'' OR ''muda'' OR ''PDSA'' OR "Plan-Do-Study-Act" OR "Plan-Do-check-Act" OR ''PDCA'' OR ''continuous quality improvement*'' OR ''CQI'' OR ''statistical quality control*'' OR ''statistical process control*'' OR ''SQC'' OR ''SPC'' OR ''control chart*'' OR ''pareto chart*'' OR "DMAIC" OR "Process mapping" OR "clinical audit*")	177,596
#1	TS=("cardiac surg*" OR "cardiac operat*" OR "cardiac preoperat*" OR "cardiac intraoperat*" OR "cardiac postoperat*" OR "cardiac perioperat*" OR "heart surg*" OR "heart operat*" OR "heart preoperat*" OR "heart intraoperat*" OR "heart postoperat*" OR "heart perioperat*")	72,983

Wiley/Cochrane Library (3)

Search	Query	Results
#4	#3 NOT ((adolescen*) OR (child*) OR (schoolchild*) OR (infant*) OR (girl*) OR (boy) OR (boys) OR (teen*) OR (youth*) OR (pediatr*) OR (paediatr*) OR (pube*)):ti,ab,kw	3
#3	#1 AND #2	146
#2	((six NEXT sigma*) OR (statistical NEXT process NEXT control) OR (root NEXT cause NEXT analys*) OR (total NEXT quality NEXT management*) OR (value NEXT stream NEXT mapping*) OR (process NEXT improvement*) OR (process NEXT assessment*) OR (TQM) OR (toyota NEXT production NEXT system*) OR (motorola NEXT production NEXT system*) OR (henry NEXT ford NEXT production NEXT system*) OR (6 NEXT sigma) OR (lean NEXT proces*) OR (lean NEXT thinking) OR (lean NEXT method*) OR (lean NEXT sigma) OR (kaizen) OR (muda) OR (PDSA) OR (Plan NEXT Do NEXT Study NEXT Act) OR (Plan NEXT Do NEXT check NEXT Act) OR (PDCA) OR (continuous NEXT quality NEXT improvement*) OR (CQI) OR (statistical NEXT quality NEXT control*) OR (statistical NEXT process NEXT control*) OR (SQC) OR (SPC) OR (control NEXT chart*) OR (pareto NEXT chart*) OR (DMAIC) OR (Process NEXT mapping) OR (clinical NEXT audit*)):ti,ab,kw	5,028
#1	((cardiac NEXT surg*) OR (cardiac NEXT operat*) OR (cardiac NEXT preoperat*) OR (cardiac NEXT intraoperat*) OR (cardiac NEXT postoperat*) OR (cardiac NEXT perioperat*) OR (heart NEXT surg*) OR (heart NEXT operat*) OR (heart NEXT preoperat*) OR (heart NEXT intraoperat*) OR (heart NEXT postoperat*) OR (heart NEXT perioperat*)):ti,ab,kw	43,809

Supplementary Table S2– PRISMA Checklist

Section/topic	#	Checklist item	Reported on page #
**TITLE**			
Title	1	Identify the report as a systematic review, meta-analysis, or both.	1
**ABSTRACT**			
Structured summary	2	Provide a structured summary including, as applicable: background; objectives; data sources; study eligibility criteria, participants, and interventions; study appraisal and synthesis methods; results; limitations; conclusions and implications of key findings; systematic review registration number.	2
**INTRODUCTION**			
Rationale	3	Describe the rationale for the review in the context of what is already known.	3
Objectives	4	Provide an explicit statement of questions being addressed with reference to participants, interventions, comparisons, outcomes, and study design (PICOS).	3
**METHODS**			
Protocol and registration	5	Indicate if a review protocol exists, if and where it can be accessed (e.g., Web address), and, if available, provide registration information including registration number.	-
Eligibility criteria	6	Specify study characteristics (e.g., PICOS, length of follow-up) and report characteristics (e.g., years considered, language, publication status) used as criteria for eligibility, giving rationale.	4
Information sources	7	Describe all information sources (e.g., databases with dates of coverage, contact with study authors to identify additional studies) in the search and date last searched.	4
Search	8	Present full electronic search strategy for at least one database, including any limits used, such that it could be repeated.	Supplementary material
Study selection	9	State the process for selecting studies (i.e., screening, eligibility, included in systematic review, and, if applicable, included in the meta-analysis).	4

Data collection process	10	Describe method of data extraction from reports (e.g., piloted forms, independently, in duplicate) and any processes for obtaining and confirming data from investigators.	4
Data items	11	List and define all variables for which data were sought (e.g., PICOS, funding sources) and any assumptions and simplifications made.	4
Risk of bias in individual studies	12	Describe methods used for assessing risk of bias of individual studies (including specification of whether this was done at the study or outcome level), and how this information is to be used in any data synthesis.	5
Summary measures	13	State the principal summary measures (e.g., risk ratio, difference in means).	-
Synthesis of results	14	Describe the methods of handling data and combining results of studies, if done, including measures of consistency (e.g., I^2) for each meta-analysis.	4

**Section/topic**	#	**Checklist item**	**Reported on page #**
Risk of bias across studies	15	Specify any assessment of risk of bias that may affect the cumulative evidence (e.g., publication bias, selective reporting within studies).	5, 8, 20
Additional analyses	16	Describe methods of additional analyses (e.g., sensitivity or subgroup analyses, meta-regression), if done, indicating which were pre-specified.	-
**RESULTS**			
Study selection	17	Give numbers of studies screened, assessed for eligibility, and included in the review, with reasons for exclusions at each stage, ideally with a flow diagram.	6-8, Figure 1, Table 1
Study characteristics	18	For each study, present characteristics for which data were extracted (e.g., study size, PICOS, follow-up period) and provide the citations.	Table 1
Risk of bias within studies	19	Present data on risk of bias of each study and, if available, any outcome level assessment (see item 12).	8, Table 2
Results of individual studies	20	For all outcomes considered (benefits or harms), present, for each study: (a) simple summary data for each intervention group (b) effect estimates and confidence intervals, ideally with a forest plot.	6-8, Table 1
Synthesis of results	21	Present results of each meta-analysis done, including confidence intervals and measures of consistency.	-
Risk of bias across studies	22	Present results of any assessment of risk of bias across studies (see Item 15).	Table 2
Additional analysis	23	Give results of additional analyses, if done (e.g., sensitivity or subgroup analyses, meta-regression [see Item 16]).	-

**DISCUSSION**			
Summary of evidence	24	Summarize the main findings including the strength of evidence for each main outcome; consider their relevance to key groups (e.g., healthcare providers, users, and policy makers).	9-12
Limitations	25	Discuss limitations at study and outcome level (e.g., risk of bias), and at review-level (e.g., incomplete retrieval of identified research, reporting bias).	12
Conclusions	26	Provide a general interpretation of the results in the context of other evidence, and implications for future research.	12
**FUNDING**			
Funding	27	Describe sources of funding for the systematic review and other support (e.g., supply of data); role of funders for the systematic review.	12
